# Combined pathological, microbiological and virological evaluation of vitreous aspirates: a retrospective evaluation of 374 vitrectomy specimens with non-neoplastic disorders

**DOI:** 10.1038/s41433-025-04047-y

**Published:** 2025-10-08

**Authors:** Mihaly Sulyok, Kristina Schmauder, Markus Schneider, Jonas Neubauer, Tina Ganzenmueller, Irina Bonzheim, Daniela Süsskind, Karl Ulrich Bartz-Schmidt, Silke Peter, Falko Fend

**Affiliations:** 1 Department of Pathology and Neuropathology, University Hospital and Comprehensive Cancer Center Tübingen, Tübingen, Germany; 2 Institute of Medical Microbiology and Hygiene, University Hospital Tübingen, Tübingen, Germany; 3 Institute for Medical Virology and Epidemiology, University Hospital Tübingen, Tübingen, Germany; 4 Centre of Ophthalmology, Eberhard Karls University of Tübingen and Comprehensive Cancer Center, University Hospital Tübingen, Tübingen, Germany

**Keywords:** Health care, Diagnosis

## Abstract

**Background:**

Intraocular infections pose substantial diagnostic challenges due to their varied aetiologies and complex inflammatory responses. The value of combining cytological, microbiological and virological assessments remains underexplored. This extensive retrospective study conducted at a German tertiary care university hospital aims to elucidate the correlation between different inflammatory patterns and detected pathogens in vitreous fluid samples.

**Methods:**

A retrospective analysis of 374 non-neoplastic vitreous samples from 353 patients was performed, integrating cytological, microbiological and virological data with final clinical diagnoses.

**Results:**

Cytological analysis showed 284 instances of lymphocytic uveitis and 76 of granulocytic endophthalmitis. Pathogens were identified in 46 out of 181 microbiologically tested samples; notable pathogens included *Staphylococcus epidermidis*, *Staphylococcus aureus*, *Streptococcus pneumoniae*, *Tropheryma whipplei*, *Candida albicans* and *Toxoplasma gondii*. Virological tests on 188 samples detected viral DNA in 42 cases, predominantly varicella zoster virus and Epstein–Barr virus, correlating well with clinical suspicions of retinitis. Interestingly, no pathogens were found in 67% of the lymphocytic uveitis cases, and multiple pathogens were detected simultaneously in seven instances, suggesting potential latent infections or reactivations. A significant pattern emerged correlating increased neutrophil counts with pathogen detection, highlighting a notable association (*p* = 0.03) in a subset analysed for both neutrophil levels and pathogen presence.

**Conclusion:**

This study offers critical insights into the epidemiology of intraocular infections in Germany, underscoring the importance of comprehensive pathological assessments. It emphasizes the diagnostic value of the underlying inflammatory patterns for predicting pathogen presence and identifies notable cases of infections, including rare pathogens like *Tropheryma whipplei*.

## Background

Uveitis is characterised by intraocular inflammation, frequently due to immune-mediated mechanisms or an infectious aetiology [1]. Despite the fact that approximately 30% of uveitis cases remain without identified aetiology, ophthalmologists need to exclude infectious uveitis as the cause of inflammation, as this directly impacts treatment options, and delay in diagnosis of infections may have catastrophic consequences [1–3]. Diagnosis of intraocular infections is often a challenging task, since numerous microorganisms may be causative, with protean manifestations [1]. The epidemiology of infectious uveitis, which comprises approximately 10–30% of all cases, varies across different regions and populations. The prevalence and distribution of certain causative pathogens can differ based on geographic location, socioeconomic factors and healthcare access [4, 5]. Of note, data on the epidemiology of infectious uveitis/endophthalmitis in Germany are scarce [6, 7].

Vitreous fluid aspirate, obtained via vitrectomy, is crucial for diagnosing infectious uveitis, enabling targeted antimicrobial therapy and improving patient outcomes. By pinpointing specific pathogens, it supports definitive diagnoses and guides treatment decisions. Additionally, analysing the pathogen profile in uveitis cases enhances epidemiological understanding, aiding in disease surveillance and management [8].

Microbiological and virological laboratory analysis involves the identification of infectious agents such as bacteria, viruses, fungi or parasites, using mainly culture-based or molecular technologies such as PCR, while cytopathological examination assesses the cellularity and cellular composition and may allow direct provisional identification of some infectious agents. Data on the relative contributions of these approaches and their potential added value, however, are sparse.

In this large, retrospective single-centre study, we analysed the association between microbiological, virological and pathological findings and provided descriptive statistics on the epidemiology of infectious uveitis and endophthalmitis at a large German tertiary care university hospital. Furthermore, it was assessed whether morphological evaluation of vitreous aspirates may help to predict the presence of infectious uveitis.

## Methods

### Patients

We identified uveitis cases from the electronic case record system (PAS-NET) of the Institute for Pathology and Neuropathology at the University of Tuebingen, using ‘Glaskörperpunktat’ (vitreous fluid aspirate) as the material type on the 10JAN2023. Cases with a pathologically confirmed diagnosis of vitreoretinal lymphoma or other malignancy were excluded. Basic demographic parameters and diagnoses were extracted, along with data on microbiological and virological findings obtained from the records of the Institutes of Medical Microbiology and Medical Virology of the University Hospital Tuebingen.

### Vitrectomy procedure

The indication for vitrectomy was based on the clinical and ophthalmological assessment by the treating physicians, usually for suspicion of infectious uveitis/endophthalmitis, lymphoma or uveitis not responding to appropriate therapy. After obtaining written consent, a 23 G vitrectomy was performed to collect a vitreous sample. The sample was taken either undiluted under air or diluted under balanced salt solution infusion, depending on the surgeon’s decision. The central vitreous was removed during the sample collection, and at the end of the surgery, the formation of peripheral holes was excluded using circular identification.

### Diagnostic procedures for bacterial, fungal and parasitological detection

Microbiological analysis of vitreous aspirates consisted primarily of culture-based diagnostics. Vitreous specimens were cultivated in nutrient broth with whole liver for unselective enrichment of microorganisms. Additionally, specimens were streaked on 5% sheep blood agar (Becton Dickinson, Heidelberg, Germany) with incubation at room air and brain-heart infusion agar plates (in-house production) with incubation under microaerophilic and anaerobic conditions. Agar plates and enrichment broth were incubated at 37 °C for 72 h. Suspected samples for fungal infections were cultivated at 30 °C on Sabouraud agar (in-house production; since 2021 Becton Dickinson, Heidelberg, Germany) for 14 days. Single colonies of grown microorganisms were identified by MALDI-TOF (Matrix-assisted laser desorption/ionisation—Time of flight) using the Microflex LT instrument (Bruker Daltonics, Germany). Culture negative specimens were analysed further by 16S-rRNA-PCR (details below) if sufficient specimen volume was available. Additional molecular diagnostics were performed depending on the differential diagnosis provided by the clinician. *Toxoplasma gondii* was detected by a conventional molecular in-house PCR assay until 2018 and afterward by a real-time PCR assay (*T. gondii* RT-PCR, Sacace Biotechnologies, Como, Italy) conducted on a Lightcycler 480 II instrument (Roche, Basel, Switzerland). *Tropheryma whipplei* was detected by a conventional molecular in-house assay until 2022 and afterward by real-time PCR (RealCycler® TRWH-GX, Progenie Molecular, Valencia, Spain). For undirected detection of bacterial DNA, an eubacterial conventional in-house PCR was performed targeting the V1-V3 region of the 16S rRNA genes (forward primer: AGAGTTTGATCCTGGCTCAG; reverse primer: TTACCGCGGCTGCTGGCAC), resulting in a fragment of approximately 500 base pairs in length. Subsequent Sanger sequencing was conducted following state-of-the-art standard diagnostic protocols. Analysis of 16S sequences was performed using the curated EzBioCloud database in the current updated version, respectively.

### Real-time PCR for virus detection

Real-time PCR assays for detection of HSV-, CMV-, VZV- or EBV-DNA were conducted on a Lightcycler 480 II (Roche) using laboratory-developed in-house PCR assays (until 2021) or the CMV, EBV or HSV-1/2&VZV Argene® assay (Biomerieux, for samples collected after 2021), depending on the differential diagnosis and assay requests by the clinician. All assays included an internal control for sufficient nucleic acid extraction and absence of PCR inhibition. Results were reported qualitatively, but the cycle threshold (Ct) values (as far as available) can be used as a measure to semi-quantitatively assess the virus concentration in a sample.

### Cytopathological analysis

Cytopathological analysis of vitreous fluid aspirates was conducted following standard procedures. The native samples were centrifuged with the Shandon Cytospin4 (4 min, speed 1100 rpm). Depending on the volume, two to four cytocentrifugates were prepared on coated glass slides. A minimum of 100 μl and a maximum of 400 μl per glass slide were pipetted, depending on the cell density of the sample (volume in μl noted on the glass slide). Following fixation, one preparation was routinely stained with May-Grünwald-Giemsa after air drying, while the remaining preparations were left to air dry. Cytopathological analysis was performed by experienced ocular cytopathologists. The slides were examined under a light microscope at various magnifications to assess cellular morphology, presence of inflammatory cells, microorganisms and any evidence of atypical or malignant cells.

### Statistical analysis

Descriptive statistics were performed using R software (version 4.0.3). A subgroup analysis of samples with both microbiological, virological and cytopathological results were performed using ordinal logistic regression to assess the the association between presence/absence of detected pathogens (as the dependent variable) and the semiquantitatively assessed neutrophilic granulocytes. Visualisation was performed using the ggplot2 package and GraphPad Prism (version 10.1.1).

The study was conducted under the ethics approval no.639/2023BO2, issued by the local ethics committee of the University Hospital Tübingen. The study was conducted in accordance with institutional regulations; all patients had given consent permitting the use of their clinical data and samples. Anonymised data and the script of statistical analysis are available from the corresponding author on request.

## Results

### Findings in cytopathological diagnostics

**Fig. 1:**
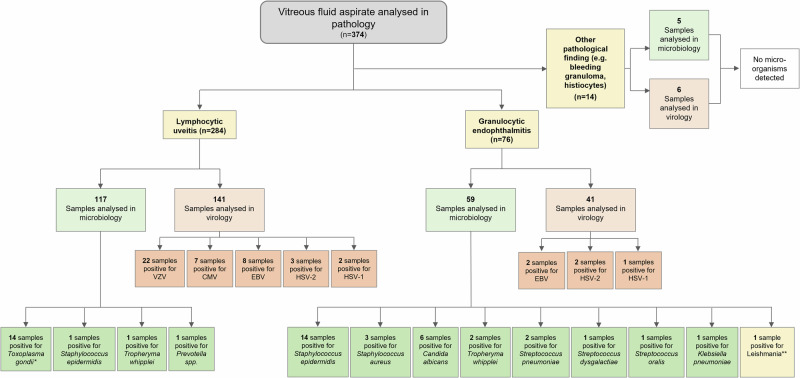
Flow chart of the analysed non-neoplastic samples displaying respective diagnostic results. * 1 case of *Toxoplasma gondii* diagnosed in pathology; ** 1 case of Leishmania diagnosed in pathology.

A total of 374 non-neoplastic vitreous fluid samples obtained from 353 patients between 1JAN2010 and 10JAN2023, were included in the analysis (Fig. 1), after exclusion of vitreoretinal lymphoma or other malignancy (*n* = 67; mostly lymphomas and one melanoma metastasis). All cases were analysed cytologically. These samples included 9 cases in which a specific pathogen was detected by cytology and also microbiologically, including 2 cases of *T. whipplei* infection and one case of *Leishmania* infection, each with granulocytic inflammation patterns, and 4 cases of intraocular toxoplasmosis with lymphocytic inflammation patterns. Moreover, 2 intraocular *Candida* infections diagnosed by pathology (one case confirmed by microbiology) displayed a granulocytic inflammation pattern.

Altogether 284 cases of lymphocytic uveitis and 76 cases of granulocytic endophthalmitis were identified. The remaining 14 samples presented intraocular bleeding, predominantly histiocytic or granulomatous inflammation as cytopathological finding.

### Pathogen detection in microbiological and virological diagnostics

Among the 181 microbiologically analysed samples, in 46 (25%) positive samples the following microorganisms were identified: *Staphylococcus epidermidis* (*n* = 15), *Staphylococcus aureus* (*n* = 3)*, Streptococcus pneumoniae* (*n* = 2), *T. whipplei* (*n* = 3), *Streptococcus oralis* (*n* = 1), *Streptococcus dysgalactiae* (*n* = 1), *Klebsiella pneumoniae* (*n* = 1), *Prevotella spp*. (*n* = 1), *Candida albicans* (*n* = 6), and *T. gondii* (*n* = 13). Additionally, 135 samples were found to be microbiologically negative (Table 1).

**Table 1:** Inflammatory patterns and detected pathogens.

	Number of Samples	%
*Samples analysed in pathology (total)*	374	100
Lymphocytic uveitis	284	75.9
Granulocytic endophthalmitis	76	20.3
Candida endophthalmitis	2	0.5
Morphologically detected aetiologies		
Toxoplasmosis (3 confirmed with PCR)	4	1.1
Candida	2	0.5
Tropheryma infection (confirmed with PCR)	2	0.5
Intraocular Leishmaniasis (confirmed with PCR)	1	0.3
Herpes simplex virus (HSV) (confirmed with PCR)	3	0.8
Other causes (e.g. intravitreal bleeding, histiocytes)	14	3.5
Samples analysed in microbiology (total)	181	48.4
* S. epidermidis*	15	4
* S. aureus*	3	0.8
* C. albicans*	6	1.6
* S. pneumoniae*	2	0.5
* T. whipplei*	3	0.8
* S. oralis*	1	0.3
* S. dysgalactiae*	1	0.3
* K. pneumoniae*	1	0.3
* Prevotella spp*.	1	0.3
* T. gondii*	13	3.5
Microbiologically Negative	135	36.1
Samples analysed in virology (total)	188	50.3
Varicella zoster virus	22	5.9
Herpes simplex virus 1	3	0.8
Herpes simplex virus 2	5	1.3
Cytomegalovirus	7	1.9
Epstein-Barr virus	10	2.7
Virologically Negative	141	37.7

Of 374 samples, a total of 188 were sent for virological assessment. Viral nucleic acids were found in 42 specimens (22%). In 93% of those samples, a lymphocytic inflammation pattern was determined by pathology. PCR detected varicella zoster virus (VZV, *n* = 22), herpes simplex virus 1 (*n* = 3), herpes simplex virus 2 (*n* = 5), cytomegalovirus (*n* = 7) and/or Epstein–Barr virus (*n* = 10) genomes. For virus positive cases, the initially suspected diagnosis upon sample submission by the treating physicians to the virology lab frequently (*n* = 18) was retinitis and/or retinal necrosis and we detected CMV-, HSV- or VZV-DNA in these samples. Among two patients with VZV-DNA detection in the vitreous fluid, preceding history of facial herpes zoster was reported. EBV was detected at low viral loads in most of the cases (see Supplementary Table 1), likely reflecting the presence of latent EBV infection of infiltrating immune cells or rather minor reactivations in seropositive individuals.

### Correlation of cytopathology, microbiology and virology

**Fig. 2:**
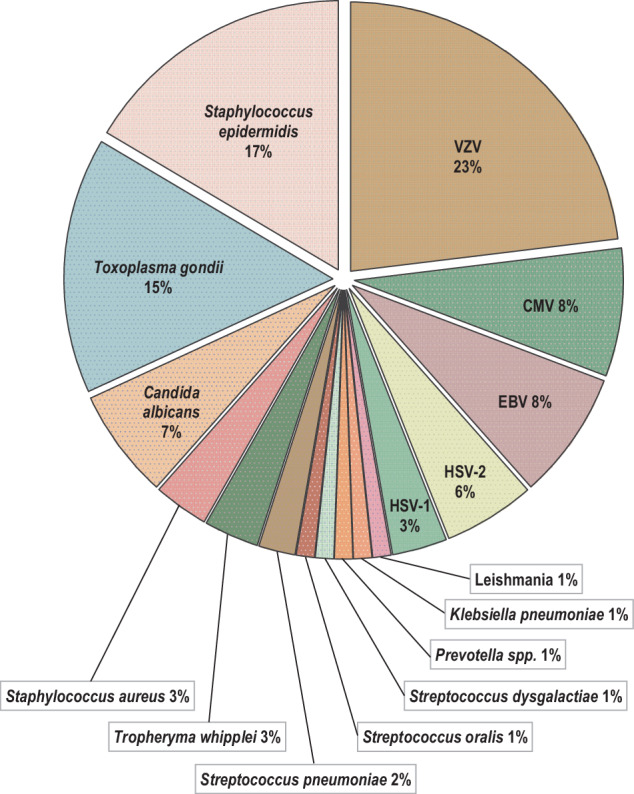
Distribution of detected pathogens in the examined cohort displayed as percentage (total *n* = 91). VZV varicella zoster virus, CMV cytomegalovirus, EBV Epstein–Barr virus, HSV-2 herpes simplex virus 2, HSV-1 herpes simplex virus 1.

In 67% (105/158) of cases of lymphocytic uveitis diagnosed by pathology and further analysed by either microbiology or virology, no evidence of an infectious agent was found. In the other 53 (33%) specimens classified as lymphocytic uveitis a causative organism could be detected. Notably, in 72% (*n*=38) of those positive cases, viruses were detected. Among the remaining cases within this subgroup, *T. gondii* (*n* = 10)*, T. whipplei* (*n* = 1)*, Prevotella spp*. (*n* = 1)*, S. epidermidis* (*n* = 1) and *C. albicans* (*n* = 1) were identified. All detected pathogens are shown in Fig. 2 and Table 1.

In total, 140 samples were assessed by both microbiology and virology diagnostics. Multiple (i.e. means two) organisms have been detected from the same specimen in seven cases (5% of this subgroup) and consisted of the combinations of any pathogen with EBV, i.e. *C. albicans* + EBV (*n* = 2), *T. gondii* + EBV (*n* = 1), VZV + EBV (*n* = 3) and CMV + EBV (*n* = 1).

We semiquantitatively categorised neutrophil abundance in this subgroup into four distinct categories: 0, 1+, 2+, and 3+. From the 140 samples, 128 were available for this subgroup analysis. Of those, the majority (100 samples; 78,1%) were classified in category 0 and in 37 out of the 100 cases (37%) pathogens were detectable. Category 1+ included 4 samples (3.1% of total samples), and in 3 of these samples, pathogens have been detected. Category 2+ comprised 4 samples (3.1% of total samples), and all of these (100% of category 2+) were tested positive for pathogens. The category with the highest neutrophil counts, 3 + , included 20 samples (15.6% of total samples), with 8 (40% of category 3+) showing pathogen presence. Statistical analysis indicated a significant association between the level of neutrophil abundance and the detection of pathogens, confirmed by chi-squared test (*p* = 0.03). The ordinal logistic regression analysis did not demonstrate a significant association between the semiquantitative categorisation of neutrophils and the likelihood of pathogen detection.

### Endophthalmitis and associated pathogens in our study

Out of the 25 cases with granulocytic endophthalmitis with positive microbiology, the clinical diagnosis was intravitreal surgical administration of medication and postoperative endophthalmitis in seven cases, mostly at external sites. Pathogens detected in those cases were *S. epidermidis, S. aureus* and *S. oralis* (Supplementary Fig. 1). Two cases were clinically classified as endogenous endophthalmitis, one due to pneumococcus endocarditis. Out of the six intraocular candida infections, two were considered endogenous endophthalmitis, panuveitis in one case, and endophthalmitis without further specification in the three remaining cases. Remarkably, all six individuals exhibited a predisposing condition, including five patients with cytotoxic or immunosuppressive therapy or radiotherapy and one with a history of intravenous drug abuse.

### Toxoplasmosis and M. Whipple in our study

**Fig. 3:**
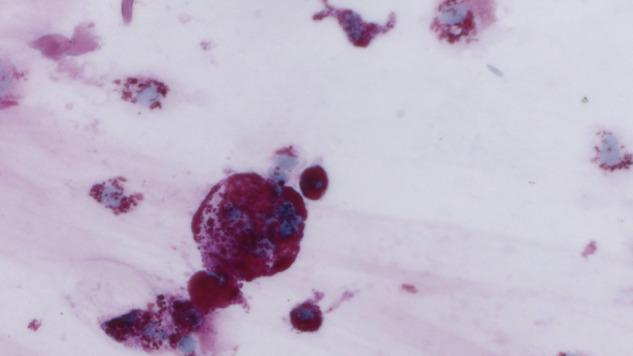
PAS positive macrophages in vitreous fluid. Granular cytoplasmic positivity is caused by massive accumulation of Tropheryma whipplei. PAS, 630x.

Regarding the 14 cases of intraocular toxoplasmosis, one patient was HIV positive, and two others were suspected of having toxoplasmosis based on clinical findings. In the three patients with intraocular Whipple’s disease (Fig. 3 and Supplementary Fig. 2), the diagnosis was not suspected clinically, but found by untargeted eubacterial 16S rRNA PCR in all patients and by PAS staining in two of them. In one of these cases, initially, CMV retinitis had been suspected clinically.

### Patient demographics

There was no significant difference between age/sex and aetiologic agent (*p* = 0.44 Kruskal Wallis test and *p* = 0.7 chi square test). The median patient age of our cohort was 66.4 years without major deviation between the different pathologic aetiologies of granulocytic endophthalmitis (median age 66.4 years) and lymphocytic uveitis (median age 66.8 years).

## Discussion

The findings of our retrospective, single-centre analysis of a large cohort of patients with vitrectomy provide valuable insights into the spectrum of aetiological agents encountered in infectious uveitis in a large tertiary care ophthalmology centre in Germany. Furthermore, the correlation between cytopathology and virological and microbiological findings underlines the importance of a synoptic diagnostic approach and provides clues for an optimal handling in non-neoplastic vitrectomy specimens.

The differential diagnosis for uveitis/vitritis and endophthalmitis includes inflammatory, infectious, immunological and neoplastic disorders, notably vitreoretinal lymphoma. Vitrectomy serves diagnostic and therapeutic roles, with specimens often analysed cytologically and tested for microbes and viruses if infection is suspected. However, systematic studies on the diagnostic value of such a multi-pronged analytical approach in non-neoplastic uveitis/endophthalmitis are sparse, and there are only limited data available regarding the aetiology of non-neoplastic uveitis and endophthalmitis in Germany.

By conducting a large retrospective single-centre study, we aimed to address this knowledge gap and provide descriptive statistics on these conditions. Our findings shed light on the prevalence and distribution of various infectious agents, such as bacteria (e.g. *S. epidermidis, S. aureus*), fungi (e.g. *C. albicans*), viruses (e.g. VZV) and parasites (e.g. *T. gondii*). Our findings are in line with the literature [9, 10], especially with regard to the dominant role of toxoplasmosis; on the other hand [11], other reportedly prevalent causes of endophthalmitis such as syphilis and tuberculosis were not identified in our series. The lack of ocular infections due to *Mycobacterium tuberculosis* in our cohort likely is a reflection of the epidemiological situation, rather than due to methodological reasons. Indeed, national tuberculosis incidence in the examined time period varied between 5.0–7.2/100.000 inhabitants and was below average in our region [12]. Moreover, tuberculous uveitis represents a rare manifestation among clinical findings in tuberculosis patients.

Infectious uveitis contributes approximately from 10 to 28% of uveitis cases; greatly depending on the geographic region and several other factors [1, 12–14]. This is roughly in line with findings in our study, where in 33% of vitreous aspirates classified as lymphatic uveitis, a pathogen could be detected. In the United States, one study identified histoplasmosis followed by viral agents (mainly HSV) and toxoplasmosis as frequently detected pathogens [13]. Another large study from India showed viral agents (HSV, VZV and CMV) as leading causes, followed by tuberculosis and only a low prevalence of toxoplasmosis (with a declining proportion from 1.69 to 0.66%). Increasing numbers of Toxocara infections, endogenous uveitis and syphilis were also reported [14]. In immunocompromised patients, opportunistic infections like invasive mould (e.g. Aspergillus), *Pneumocystis*, atypical mycobacteria may also cause devastating intraocular infections.

Among cases of granulocytic endophthalmitis where an infectious pathogen could be identified in our study, some were associated with antecedent intravitreal surgical procedures, emphasising the need for vigilant aseptic techniques during and following such interventions [15]. In this subpopulation, coagulase-negative Staphylococci were notably prevalent. The presence of endocarditis-related Pneumococcus and other endogenous sources, such as systemic candida infections, further illustrates the rare but potential origins of ocular infections by septic spreading. Proactive assessment for ocular involvement has to be taken into account when diagnosing systemic infections and concomitant visual impairment [16, 17]. Intraocular Candida infections present a unique diagnostic challenge. This underscores the necessity for comprehensive clinical assessments, including consideration of underlying systemic conditions.

Interestingly, in three cases, we detected the bacterium *T. whipplei*, responsible for a rare systemic condition that predominantly affects the gastrointestinal tract but can manifest in various extraintestinal sites, including the eyes [18]. The pathophysiology of Whipple-uveitis remains poorly understood, but it is hypothesised that bacterial infiltration and subsequent immune response contribute to ocular inflammation. Epidemiological data from Germany indicate an incidence of Whipple’s disease at approximately 0.5–1.0 cases per million per year, with uveitis being a less commonly reported manifestation [19]. In a retrospective study analysing German patient data, ocular involvement was observed in about 10% of Whipple’s disease cases [20]. Our findings underscore the importance of Whipple uveitis. Early diagnosis and treatment are crucial to prevent irreversible ocular damage and systemic complications [21]. Future research should focus on elucidating the molecular mechanisms driving ocular involvement in Whipple’s disease and optimising therapeutic strategies to improve patient outcomes.

Our study emphasises the importance of combined pathological, virological and microbiological examination of vitreous aspirates in the diagnosis of intraocular infections. The absence of granulocytes in vitreous aspirate specimens was identified as a highly indicative marker for a negative bacteriological or fungal result, thereby assisting in the differentiation between bacterial/fungal and non-bacterial/non-fungal causes of endophthalmitis. In contrast, a pattern of granulocytic inflammation identified by histopathology resulted in the detection of causative bacterial or fungal organisms in only around 50% of cases, either by culture or by PCR within our cohort (27 cases with bacterial pathogens, 5 cases with Candida, 5 cases with viruses). Neutrophilic granulocytes play a crucial role in averting the expansion of mainly bacterial or fungal infections and are seen frequently in cases of postoperative endophthalmitis [22–24]. However, as findings from this cohort reveal, herpes simplex virus 1 or 2 infections can lead to invasion of granulocytes as well. A retrospective cytopathologic study of acute retinal necrosis cases also found polymorphonuclear cells alongside lymphocytes and macrophages in nearly one-third of vitrectomy samples in confirmed HSV or VZV infection [25]. This indicates both innate and adaptive immune activation, highlighting the diagnostic challenge of granulocyte-rich inflammation in viral uveitis. Moreover, recruitment of neutrophilic granulocytes in sterile tissue injury or non-infectious inflammatory diseases are commonly known and might play a confounding role when using granulocytic inflammation as a marker for bacterial or fungal infections [26, 27].

Concurrent high numbers of neutrophils (3+) appear to reduce successful pathogen detection in our cohort and are most likely reflecting intensified inflammation processes resulting in degradation of vital organisms, as well as DNA digestion, aggravating thereafter cultural and molecular diagnostics. Moreover, purulent inflammation often necessitating dilution of specimens before vitreous extraction reduces pathogen load and therefore detection sensitivity. Indeed, French data on postoperative bacterial endophthalmitis reveal culture-positive detection of microorganisms in up to 64.7% in directly obtained, undiluted vitreous aspirate specimens [28]. Obtained sample volume remains a further known key factor for sensitive pathogen detection, which can be challenging considering the anatomically small extent within the ocular bulb. To further address this diagnostic issue in sensitive pathogen detection, untargeted molecular strategies like evolving Next-generation sequencing technologies could be of help and have already shown to be of beneficial use in uncovering intraocular infections, which however, need further contextual evaluation in the future [29].

While the majority of cases with lymphocytic uveitis did not exhibit an infectious aetiology, our study revealed cases with, probably, reactivating virus infections were identified. These were predominantly caused by VZV, followed by CMV and HSV infections, which is in line with the literature [30, 31]. In a number of vitreous specimens, EBV DNA was detected. However, high Ct-values in EBV real-time PCR suggest very low viral loads and likely represent the presence of rare latently infected lymphocytes in uveitis of other cause, rather than active disease. Notably, EBV was frequently detected together with other pathogens, e.g. concurrent toxoplasmosis. While minor EBV reactivation is possible, especially in a single case with a Ct-value of 24.8, EBV uveitis is only rarely reported in the literature [32]. This highlights that virus detection, especially in the case of EBV, does not necessarily indicate causality.

Besides viral infections, *T. gondii* was identified as a frequent pathogen presenting with lymphatic infiltration of the vitreous body. 1 out of 14 of the discordant lymphocytic uveitis cases with positive microbiological result and without identified pathogen in the pathological report initiated reviewing of the pathology slides and led to retrospective identification of a Toxoplasma bradycyst. This highlights the importance of considering infectious causes in a multidisciplinary fashion, even in cases presenting as subacute, suspectedly non-infectious uveitis.

Vitreous opacities encompass a broad diagnostic range, from benign to life-threatening conditions. As emphasised by Coupland et al. [33], accurate aetiological classification and diagnostic yield from vitreous biopsies depend critically on proper specimen handling, timely fixation, and the application of specialised staining and molecular techniques. A prospective study using appropriate mucolytic pre-treatment and fixation in PreservCyt demonstrated that diagnostic material could be obtained in over 90% of vitreous biopsies, enabling both morphological and immunocytochemical characterisation of inflammatory and neoplastic processes [34].

## Conclusions

While lymphocytic uveitis cases were either related to the detection of viral pathogens and *T. gondii* or lacked an infectious aetiology, granulocytic patterns in the vitreous specimens indicated rather bacterial infections. Combining pathological, microbiological and virological examinations of vitreous aspirates offers a valuable diagnostic approach for intraocular infections. In a small but significant subset, *T. whipplei* was identified as the causative organism, underscoring the importance of considering this pathogen in diagnostic evaluations. This study contributes important epidemiological insights and highlights the significance of comprehensive analyses in the evaluation of non-neoplastic vitrectomy specimens.

## Summary

### What was known before

- Intraocular infections pose significant diagnostic challenges due to their diverse aetiologies and complex inflammatory responses.
- Data correlating cytological findings with detected pathogens in vitreous fluid are limited, impacting diagnostic precision.

### What this study adds

- Provides a comprehensive analysis of 374 non-neoplastic vitreous samples from 353 patients, focusing on both microbiological and virological assessments.
- Identifies significant pathogens, including *T. whipplei* and *T. gondii*, in a subset of samples
- Demonstrates an association between increased neutrophil counts and pathogen detection, enhancing diagnostic accuracy for intraocular infections.

## Supplementary information

Supplementary Figure 1.

Supplementary Figure 2.

Supplementary Table

Supplementary Figure Legends

## Data Availability

The datasets analysed during the current study are not publicly available due to privacy and ethical restrictions but are available from the corresponding author upon reasonable request in anonymised form.
