# Supplementary material for: Combined pathological, microbiological and virological evaluation of vitreous aspirates: a retrospective evaluation of 374 vitrectomy specimens with non-neoplastic disorders
Source: Eye (Lond). 2025 Oct 8;39(18):3262–8. doi: 10.1038/s41433-025-04047-y (PMC12669569; doi:10.1038/s41433-025-04047-y)
Supplement: Supplementary file 1 — Supplementary Figure 1. [file 41433_2025_4047_MOESM1_ESM.pdf]

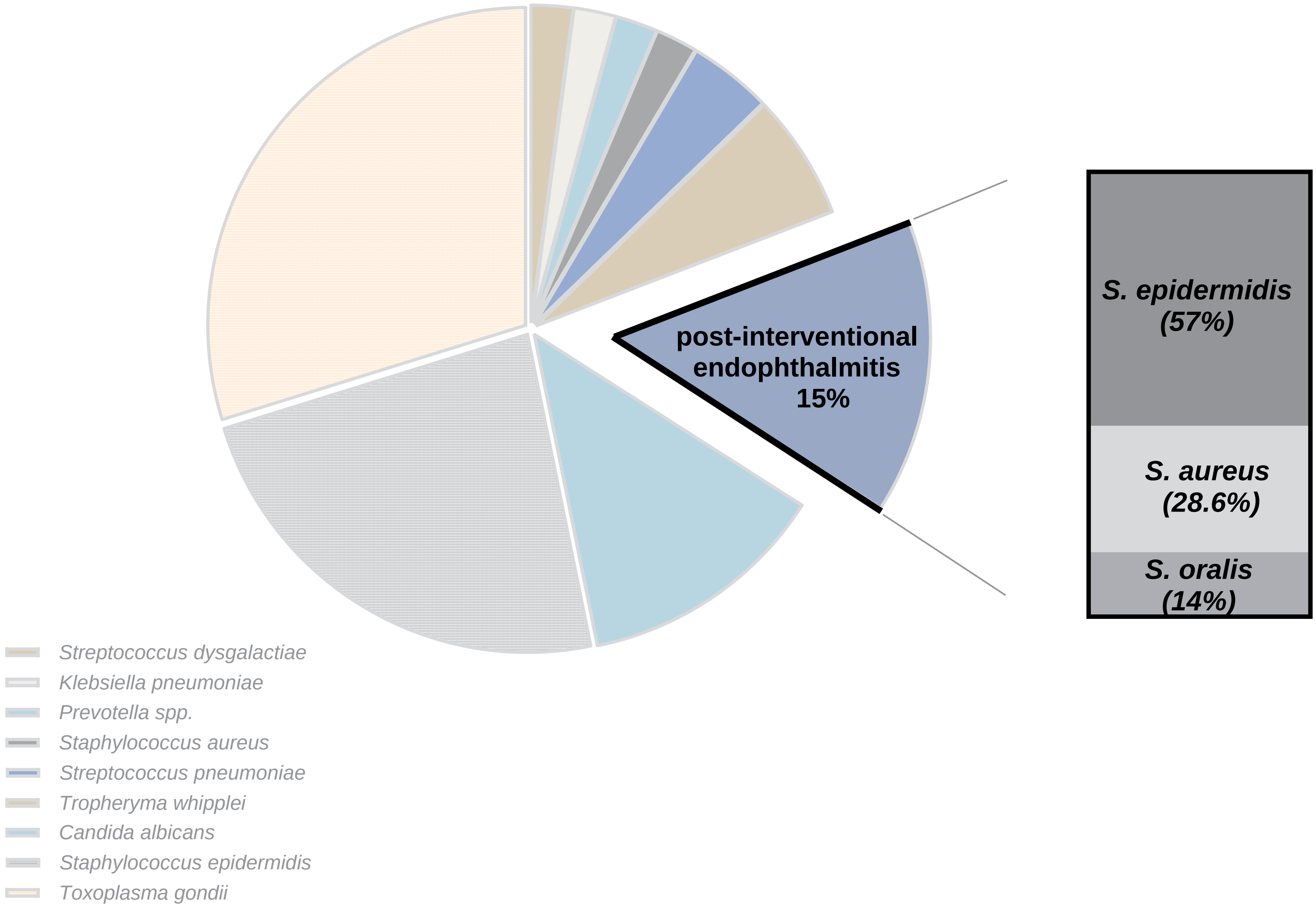
post-interventional endophthalmitis 15%
S. epidermidis (57%)
S. aureus (28.6%)
S. oralis (14%)
Streptococcus dysgalactiae
Klebsiella pneumoniae
Prevotella spp.
Staphylococcus aureus
Streptococcus pneumoniae
Tropheryma whipplei
Candida albicans
Staphylococcus epidermidis
Toxoplasma gondii
