# Supplementary figures and images for: Combined pathological, microbiological and virological evaluation of vitreous aspirates: a retrospective evaluation of 374 vitrectomy specimens with non-neoplastic disorders
Source: Eye (Lond). 2025 Oct 8;39(18):3262–8. doi: 10.1038/s41433-025-04047-y (PMC12669569; doi:10.1038/s41433-025-04047-y)

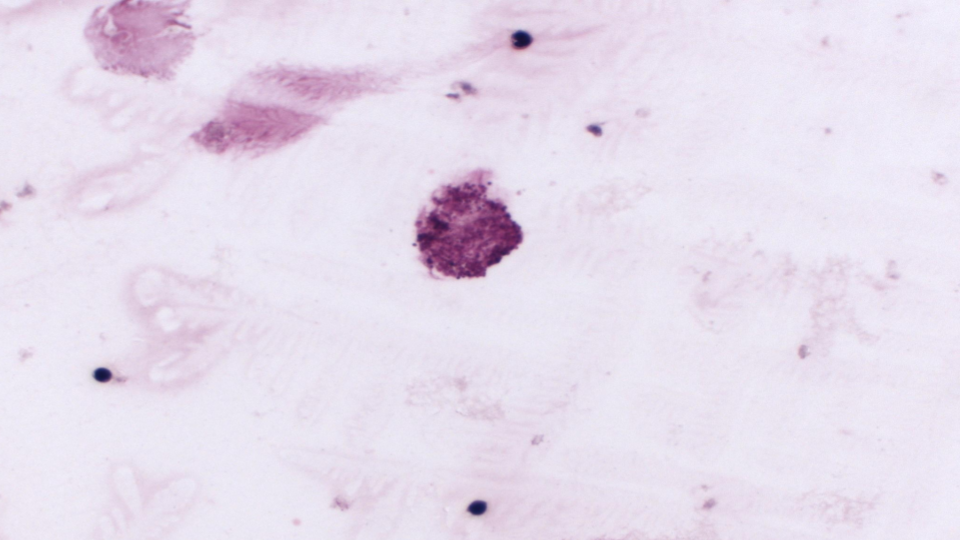

Supplement: Supplementary file 2 — Supplementary Figure 2. [file 41433_2025_4047_MOESM2_ESM.tif]
