# Supplementary material for: Combined pathological, microbiological and virological evaluation of vitreous aspirates: a retrospective evaluation of 374 vitrectomy specimens with non-neoplastic disorders
Source: Eye (Lond). 2025 Oct 8;39(18):3262–8. doi: 10.1038/s41433-025-04047-y (PMC12669569; doi:10.1038/s41433-025-04047-y)
Supplement: Supplementary file 3 — Supplementary Table [file 41433_2025_4047_MOESM3_ESM.docx]

Supplementary Table 1.

| **Diagnosis Pathology** | **Pathgogen detected** | **EBV CT Value** | **Clinical information** |
| --- | --- | --- | --- |
| lymphocytic uveitis | VZV/EBV | 24,8 | Herpes Zoster |
| lymphocytic uveitis | EBV/no data Mibi | 31 | *no data* |
| lymphocytic uveitis | EBV/Toxo | 32 | Uveitis |
| granulocytic endophtalmitis | EBV/Candida | 33,9 | Uveitis |
| lymphocytic uveitis | EBV | 35,3 | Uveitis |
| lymphocytic uveitis | EBV | 35,9 | *no data* |
| lymphocytic uveitis | CMV/EBV | 36,3 | CMV-Retinitis |
| lymphocytic uveitis | VZV/EBV | 37 | *no data* |
