# Supplementary material for: Combined pathological, microbiological and virological evaluation of vitreous aspirates: a retrospective evaluation of 374 vitrectomy specimens with non-neoplastic disorders
Source: Eye (Lond). 2025 Oct 8;39(18):3262–8. doi: 10.1038/s41433-025-04047-y (PMC12669569; doi:10.1038/s41433-025-04047-y)
Supplement: Supplementary file 4 — Supplementary Figure Legends [file 41433_2025_4047_MOESM4_ESM.docx]

Supplementary Figure 1. Distribution of all detected bacterial pathogens in the examined cohort (n=47) with subanalysis of cases with known post-interventional endophthalmitis displayed as percentage.

Supplementary Figure 2. Different cytological manifestations of Toxoplasma gondii infection. A: bradycyst in vitreous fluid. HE, 630x
